# The Impact of Time from Diagnosis to Operating Room on Functional Outcomes in Patients with Traumatic Brain Injury: A Retrospective Study

**DOI:** 10.1177/08977151251366985

**Published:** 2025-09-04

**Authors:** Cheewin Khawprapa, Sasithorn Khawprapa

**Affiliations:** ^1^ Department of Neurosurgery, Sakon Nakhon Hospital, Sakon Nakhon, Thailand.; ^2^ Department of Physical Medicine and Rehabilitation, Sakon Nakhon Hospital, Sakon Nakhon, Thailand.

**Keywords:** functional outcomes, traumatic brain injury, timing of surgery

## Abstract

This study compares the functional outcomes of post-traumatic neurosurgical patients who underwent surgery within 4 h of diagnosis with those who underwent surgery >4 h after diagnosis. A retrospective analysis was conducted on patients who underwent traumatic neurosurgery at Sakon Nakhon Hospital between 2018 and 2024. The study included 164 patients, divided into two groups of 82 patients each. Group 1 underwent surgery within 4 h of diagnosis, while Group 2 underwent surgery after 4 h. The Glasgow Outcome Scale—Extended scores at 3 months post-operatively were significantly more favorable in Group 1 compared with Group 2 (*p* = 0.011). Additionally, the Barthel Index at 1 month and 3 months post-operatively was significantly higher in Group 1 (*p* = 0.044, 0.007, respectively). The findings suggest that early surgical intervention within 4 h of diagnosis leads to better functional outcomes in patients with traumatic brain injury.

## Introduction

Traumatic brain injury (TBI) is a significant global health problem that often results in long-term disability and death.^1,2^ A survey revealed that 69 million TBI cases were reported worldwide, highlighting the need to develop systematic, effective treatments.^3^ The severity of TBI can range from mild to severe, depending on the mechanism, such as falls, sports injuries, or traffic accidents. Owing to the brain’s complexity and vital functions, prompt surgical treatment is crucial for optimal outcomes.^4,5^

The pathophysiology of TBI can be broadly divided into primary and secondary brain injuries. Primary brain injuries result from direct impacts on the entire brain and include diffuse brain injury, cortical contusion, intracranial hemorrhage, and penetrating brain injury. Secondary brain injury is caused by the biochemical deterioration of neurons following cerebral metabolic dysfunction, excitotoxicity, and oxidative stress. These injuries result in elevated intracranial pressure (ICP). Cerebral blood flow insufficiency subsequently occurs, ultimately leading to cerebral hypoxia.^6,7^

Delays in obtaining emergency surgery erode treatment outcomes for traumatic brain injuries. Neurosurgical delays can lead to neurological deterioration and thus increase the likelihood of adverse long-term outcomes, including mortality.^8–12^ Additionally, a previous study has demonstrated that surgical intervention within 12 h of TBI can reduce mortality rates.^13^ Albanèse et al. reported that early neurosurgical intervention of hemorrhagic TBI cases with a Glasgow Coma Scale (GCS) of <6 and cerebral herniation resulted in 25% of patients successfully recovering and reintegrating into society within a year.^14^

Notably, despite undergoing surgical evacuation within 4 h of injury, patients with acute subdural hematoma demonstrate a significantly poorer outcome.^15^ Furthermore, in severe TBI cases, the time to surgical intervention did not show a significant correlation with mortality rates.^16^

Although previous meta-analyses have compared the long-term outcomes of early versus late decompressive craniectomy in patients with moderate-to-severe TBI, the findings did not demonstrate a significant improvement in long-term outcomes associated with early intervention.^12^ Moreover, in previous studies, the impact of early intervention on long-term outcomes was variable. Therefore, this study aimed to compare the impact of the time from diagnosis to the operating room on the functional outcomes of patients with TBI and thus to more effectively develop head injury protocols.

## Methods

This retrospective cohort study included patients with TBI who underwent surgery and were admitted to the inpatient department of Sakon Nakhon Hospital between 2018 and 2024. All patients were over 18 years of age and had undergone craniotomy or craniectomy. Patients with inadequate diagnostic and operative data were excluded from the study.

The patients with TBI were transferred to the emergency department (ED) and underwent an emergency computed tomography (CT) brain for a definitive diagnosis. The patients were divided into two groups using a cutoff point of 4 h from the definitive diagnosis to the operating room: the early group, who underwent neurosurgery within 4 h of diagnosis, was labeled Group 1, and the late group, who underwent surgery beyond 4 h after (brain [CT] scan), was labeled Group 2.

Demographic information, including patient sex, age, mechanism of injury, GCS score in the ED, and time from the scene to the ED, was collected. Staff radiologists recorded the findings and time of starting the CT scans in the radiographical reports. In the post-operative period, complications were reported, including the duration of mechanical ventilation, length of intensive care unit (ICU) stay, and hospital stay.

The functional outcomes of the patients with TBI in this study were evaluated based on the Glasgow Outcome Scale—Extended (GOSE) scores and Barthel Index at 1 and 3 months post-operatively. Patients with GOSE scores of <4 were described as having unfavorable outcomes, and those with a GOSE score of ≥4 were allocated to favorable outcomes.^17,18^ Likewise, patients with a higher Barthel Index scale indicated a better ability to self-reliance.^19^

### Statistical analysis

We analyzed patient demographic data using descriptive statistics. The chi-square test was used to compare the GOSE scores and complications between the two groups. An independent-samples *t*-test was used to assess the differences between the groups in the Barthel Index, duration of mechanical ventilation, and duration of hospital stay, including the time spent in the ICU.

## Results

### Patient population

The study divided 164 patient cases into two groups, each with the same number. In Group 1, 87.8% of patients (82 cases) were male, while 72% of patients in Group 2 were male.

The average patient age was 45.1 ± 17.4 years in Group 1 and 48.23 ± 16.5 years in Group 2. The respective GCS scores of patients evaluated in the ED for each group were 8.5 ± 4.5 and 9.6 ± 4.5. The most common cause of TBI was motorcycle crashes. Regarding brain CT evaluation, over half of the patients in both groups had <10 mm of midline shift on brain imaging. In addition, approximately half of both groups had subdural hematoma. The time from diagnosis to the operating room in Group 1 was 2.5 ± 1.12 h, while Group 2 underwent surgical treatment 18.26 ± 27.89 h after diagnosis. The other baseline characteristics are presented in Table 1.

**Table 1.** Participants Baseline Characteristics

	Early intervention (within 4 h) *N* = 82	Late intervention (more than 4 h) *N* = 82	*p* Value
Age (years), mean ± SD	45.18 ± 17.4	48.23 ± 16.5	0.25
Male, *N* (%)	72 (87.8)	59 (72.0)	0.011^*^
Mechanism of injury, *N* (%)			
Motorcycle crash	37 (45.1)	42 (51.2)	0.435
Car accident	11 (13.4)	6 (7.3)	0.200
Fall	31 (37.8)	24 (29.3)	0.247
Assault	1 (1.2)	7 (8.5)	0.030^*^
GCS (admission), mean ± SD	8.5 ± 4.5	9.6 ± 4.5	0.138
Midline shift, *N* (%)			
<10 mm	48 (58.5)	59 (72.0)	0.072
10–20 mm	29 (35.4)	22 (26.8)	0.317
>20 mm	5 (6.1)	1 (1.2)	0.098
Lesion, *N* (%)			
Epidural hematoma	29 (35.4)	21 (25.6)	0.175
Subdural hematoma	56 (68.3)	40 (48.8)	0.011^*^
Subarachnoid hematoma	9 (11.0)	14 (17.1)	0.261
Brain contusion	14 (17.1)	24 (29.3)	0.064
Depressed skull fracture	3 (3.7)	7 (8.5)	0.192
Diagnosis to operating room (hour), mean ± SD	2.50 ± 1.12	18.26 ± 27.89	9.6751
Type of operation, *N* (%)			
Craniotomy	30 (36.6)	23 (28.0)	0.243
Craniectomy	52 (63.4)	59 (72.0)	0.319

^*^ Indicates statistically significant difference (*p* < 0.05).

GCS, Glasgow Coma Scale; SD, standard deviation.

### Functional outcomes after surgery

Group 1 revealed a significantly more favorable GOSE score (5–8) at 3 months post-operatively than Group 2 (*p* = 0.011); however, there was no statistically significant difference at 1 month post-operatively (*p* = 0.618). Furthermore, the Barthel Index at the 1-month and 3-month periods in Group 1 was significantly higher than that in Group 2 (*p* = 0.044 and 0.007, respectively) (Table 2).

**Table 2.** Functional Outcome

	Early intervention (within 4 h) *N* = 82	Late intervention (more than 4 h) *N* = 82	*p* Value
GOSE 1-month median (IQR)	7 (2–8)	7 (2–8)	0.081
GOSE 3-month median (IQR)	8 (3–8)	7 (2–8)	0.022^*^
GOSE favorable score (5–8) at 1 month, *N* (%)	56 (54.9%)	46 (45.1%)	0.618
GOSE favorable score (5–8) at 3 months, *N* (%)	58 (54.2%)	49 (45.8%)	0.011^*^
Barthel Index 1 month, mean ± SD	78.26 ± 31.10	69.36 ± 35.52	0.044^*^
Barthel Index 3 months, mean ± SD	86.52 ± 29.34	74.57 ± 36.74	0.007^*^

^*^ Indicates statistically significant difference (*p* < 0.05).

GOSE, Glasgow Outcome Scale—Extended; IQR, interquartile range; SD, standard deviation.

Table 3 presents a comparison of complications between the two groups, highlighting that the early surgical intervention group had a significantly lower incidence of ventilator-associated pneumonia (VAP), syndrome of inappropriate antidiuretic hormone secretion (SIADH), and shorter ICU stays. However, there were no significant differences in mortality rates, duration of mechanical ventilation, or length of hospital stay between the groups.

**Table 3.** Comparison of Complications Between Early and Late Interventions

	Early intervention (within 4 h) *N* = 82	Late intervention (more than 4 h) *N* = 82	*p* Value
Surgical wound infection, *N* (%)	1 (1.2)	3 (3.7)	0.31
Ventilator-associated pneumonia, *N* (%)	20 (24.4)	36 (43.9)	0.008^*^
SIADH, *N* (%)	0	4 (4.9)	0.04^*^
Death, *N* (%)	13 (15.9)	12 (14.6)	0.82
Length of mechanical ventilation (more than 96 h), *N* (%)	28 (40.6)	36 (51.4)	0.199
Length of ICU stay (day), mean ± SD	7.80 ± 6.47	10.63 ± 8.54	0.045^*^
Length of hospital stay (day), mean ± SD	13.80 ± 10.31	16.30 ± 12.01	0.156

^*^ Indicates statistically significant difference (*p* < 0.05).

ICU, intensive care unit; SD, standard deviation; SIADH, syndrome of inappropriate antidiuretic hormone secretion.

## Discussion

This retrospective study aimed to determine whether the interval between diagnosis and neurosurgical intervention impacts post-operative functional outcomes in patients with TBI. The results indicated that patients who underwent surgery within 4 h of diagnosis had significantly better GOSE scores at 3 months and more favorable GOSE outcomes. Additionally, when considering the Barthel Index, the group that underwent surgery within 4 h had significantly higher scores than the other group. This is consistent with a prior study focusing on early bilateral decompressive craniectomy, which was related to better outcomes.^20^

Additionally, in the case of decompressive craniectomy within 4 h of trauma, there were significantly better Glasgow Outcome Scale scores at 6 months compared with patients with delayed surgical decompression.^21^ Previous studies suggest that early neurosurgical interventions can help control ICP effectively and improve functional outcomes.^20^

The results indicated that patients with TBI who received early neurosurgical interventions exhibited a significantly lower incidence of VAP than those who underwent late-onset surgery. This reduction may be attributed to the improvement in neurogenic pulmonary edema, a critical respiratory complication that occurs in ∼20% of severe head injury cases. Such improvement, caused by surgically decreasing ICP,^22^ contributed to shortening mechanical ventilator use, which is associated with a low occurrence of VAP and shortening ICU spans. Moreover, the results also showed a significantly shorter length of ICU stay in the early surgical intervention group than in the late group, accounting for 7.8 and 10.6 days, respectively.

Several studies have elucidated the mechanisms underlying VAP and hospital-acquired pneumonia and their impact on functional outcomes in patients with TBI. These studies have identified that pathological abnormalities in the lungs lead to ineffective gas exchange, which plays a critical role in the development of hypoxemia, which, in turn, results in decreased cerebral oxygenation. Consequently, cerebral hypoxemia impairs brain function, ultimately leading to adverse outcomes.^23–25^

Gregson et al. conducted a study on patients with TBI and found that surgical evacuation within the first 12 h after brain injury significantly reduced the mortality rate in patients with TBI.^13^ This observation differs from the findings of the present study, which showed no difference in mortality rates between the two groups. This discrepancy may be due to differences in the brain pathologies studied. Gregson et al. specifically focused on patients with traumatic intracerebral hemorrhage, whereas our study included patients with other brain pathologies, namely epidural hematoma and subdural hemorrhage. This may be a potential factor resulting in variations in the mortality rate.

The limitation of the current study is that patients with comorbid multiple traumas were not excluded from the study, which may have influenced patient GOSE scores and mortality in both groups. Additionally, since the ICP monitoring device, which helps neurosurgeons manage TBI cases, is not available in our hospital, it may impact functional outcomes.

## Conclusion

Initiating neurosurgical operations within 4 h of diagnosis benefits patients with TBI, with significantly better 3-month GOSE scores and improved Barthel Index results at 1 and 3 months. Additionally, early surgical intervention reduces the incidence of VAP and shortens the length of stay in the ICU.

## Transparency, Rigor, and Reproducibility Summary

This study was not formally registered because it was a retrospective analysis of existing data. The Ethics Committee for Sakon Nakhon Hospital reviewed and approved the research. This study clearly describes the methods and procedures used, including patient selection, data collection, and outcome measurement. Patients were selected based on their admission to the inpatient department of Sakon Nakhon Hospital between 2018 and 2024, and all were over 18 years of age and had undergone craniotomy or craniectomy. The study details the inclusion and exclusion criteria, the diagnostic tools used (emergency CT brain scans), and the statistical methods applied (chi-square test and independent-samples *t*-test). The robustness of the study design is emphasized through the steps taken to minimize bias, such as the division of patients into two groups based on the time from diagnosis to the operating room. The study acknowledges limitations, such as the inclusion of patients with comorbid multiple traumas and the lack of ICP monitoring devices. These factors were addressed by thorough data analysis and comparison of baseline characteristics. The experimental procedures and data analysis are detailed to ensure reproducibility. Raw data, including demographic information, GCS scores, and functional outcomes (GOSE and Barthel Index), are available upon request from the corresponding author. The study also discusses efforts to validate findings through comparison with previous studies.

## Data Availability

Data are available upon request from the corresponding author.

## Abbreviations Used

CT: Computed Tomography
ED: Emergency Department
GCS: Glasgow Coma Scale
GOSE: Glasgow Outcome Scale—Extended
ICP: Intracranial Pressure
ICU: Intensive Care Unit
IQR: Interquartile Range
SD: Standard Deviation
SIADH: Syndrome of Inappropriate Antidiuretic Hormone Secretion
TBI: Traumatic Brain Injury
VAP: Ventilator-Associated Pneumonia
